# International Trade of CITES Listed Bird Species in China

**DOI:** 10.1371/journal.pone.0085012

**Published:** 2014-02-19

**Authors:** Linlin Li, Zhigang Jiang

**Affiliations:** 1 Key Laboratory of Animal Ecology and Conservation Biology, Institute of Zoology, Chinese Academy of Sciences, Beijing, China; 2 University of Chinese Academy of Sciences, Beijing, China; 3 Endangered Species Scientific Commission of People’s Republic of China, Beijing, China; Biodiversity Insitute of Ontario - University of Guelph, Canada

## Abstract

Commercial trade of wild birds may devastate wild bird populations. Convention on International Trade in Endangered Species of Wild Fauna and Flora (CITES) controls the trade of wild species listed in its appendices to avoid these species being threatened by international trade. China used to be one of the major trading countries with significant bird trade with foreign countries; on the other hand, China is a country with unique avian fauna, many Important Bird Areas and critically endangered bird species. What is the role of the country in world wild bird trade? What kind of insights can we extract from trade records for improving future management of wild bird trade in the country? We retrieved and analyzed international trade records of the CITES listed bird species of China from 1981 to 2010 from the CITES Trade Database maintained by United Nations Environment Program and World Conservation Monitoring Centre (UNEP-WCMC). We found that: (1) International trade of live birds in China peaked during the late 1990s, then decreased to the level before the surge of trade in a few years, the trade dynamics of wild birds may be affected by governmental policy and the outbreak of avian influenza during the period. (2) Most frequently traded CITES Appendix listed birds in China were parrots, most of which were exotic species to the country. (3) Birds were mainly traded for commercial purpose. Exotic birds in trade were mainly captive-bred while the most Chinese birds traded internationally were captured from the wild. Since many bird species in international trade are threatened to extinction, China should take stricter measures on importing of wild-captured birds and should collaborate with the countries of original in the international bird trade to avoid unsustainable harvesting of wild birds. It is urgent for China to carry out population surveys on those domestic bird species once in significant international trade and to make better conservation decisions based on population status of those birds. In addition, more scientific researchers should be encouraged to participate in the wildlife trade management to avoid misreporting trade data or biased analyses of the trade records.

## Introduction

Biodiversity declines are mainly caused by habitat destruction, over-exploitation, exotic species invasion and global climate change while wildlife trade has a significant influence on biological resources exploitation [1]. Unregulated trade may lead to over-exploitation and bring species to extinction [2], [3]. The Convention on International Trade in Endangered Species of Wild Fauna and Flora (CITES) is an international treaty aiming at regulating international trade of wildlife. International trade of wild species listed in CITES Appendices are controlled for avoiding those species threatened by international trade. The convention came into effect in 1975 and China ratified CITES in 1981 (http://cites.org.cn/index.php?id=102). CITES manages international trade of specimens of species of wild fauna of flora including “living or dead and any readily recognizable part or derivative” in relation to the species listed in its appendixes [4]. National CITES management authority should report the sources and trade purposes of each trade of CITES Appendixes listed species. Wild-captured species or commercial trade of CITES Appendixes listed species are in more strict control than others [4].

There are more than 10,000 bird species living in the world with nearly half of them being used directly by human and about 1,500 of them are listed in CITES Appendices [5]. Some researchers have calculated Red List Indices (RLIs) of birds and concluded the threats to birds worldwide had been persisted since 1988 [6]. Pressures from international trade on birds are mounting since the trade volume of trade of birds as pets remains high [7], [8]. China is a country with the largest consumer population in the world and used to be a state which has significant trade in birds; at the same time, China also has a large number of threatened bird species classified in the IUCN Red List [9], [10], [11], [12]. One hundred fifty-six Chinese bird species are listed in CITES Appendices. What is the role of China in world wild bird trade? What insight can we extract from the management of international bird trade by the country? In this paper, we analyzed international trade of CITES Appendix listed birds in China in terms of the major bird species in trade, main trade countries, volume in trade, the trends of trade and trade purposes. The results will not only be useful for bird international trade management but also useful for bird resources monitoring, restoration and conservation.

## Methods

The CITES Trade Database is managed by United Nations Environment Program and World Conservation Monitoring Centre (UNEP-WCMC) on behalf of the CITES Secretariat. It contains more than 10 million records of wildlife trade and 50,000 scientific names of taxa listed by CITES from 1975 to date [13]. In the CITES Trade Database, bird trade refers to international trades of live birds, bodies, feathers, eggs, specimens, etc. These records are derived from the annual reports submitted to the CITES Secretariat by the parties of CITES. Specimen sources are divided into nine categories: A (Artificially propagated plants), C (Captive-bred animals), D (Appendix-I animals bred in captivity or Appendix-I plants artificially propagated for commercial purposes), F (Animals born in captivity including F1 and subsequent), I (Confiscated or seized specimens), O (Pre-Convention specimens), R (Specimens of animals reared in a controlled environment), U (Source unknown which must be justified), W (Specimens taken from the wild). Trade purposes include B (Breeding in captivity or artificial propagation), E (Educational), G (Botanical garden), H (Hunting trophy), L (Law enforcement/judicial/forensic), M (Medical including biomedical research), N (Reintroduction or introduction into the wild), P (Personal possession), Q (Circus and traveling exhibitions), S (Scientific), T (Commercial), and Z (Zoo) [13]. Because of the limitations in scientific and management capability of different countries, the records may not be exactly consistent with real trades [13]. In addition, units of eggs, feathers and other specimens are missing or inconsistent between import and export countries. Therefore, we took out these records of eggs and feathers due to they were only small number of the trade records. Then, we analyzed live birds in international trade of China from 1981 to 2010. The CITES Trade Database records species’ trade information as detailed as possible. We summarized the trade volume of different trade purposes and specimen sources to find out which bird species may be affected by legal international trade.

## Results

### An Overview of International Live Bird Trade in China

**Figure 1:**
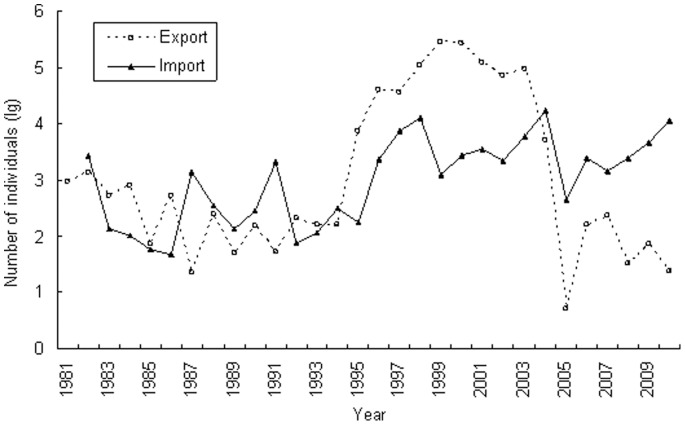
Export and import of live birds in China from 1981 to 2010.

During the period from 1981 to 2010, the total live birds exported from China exceeded one million individual birds while more than 80,000 individual birds imported to China during the same period (Figure 1). Quantity of bird export from China fluctuated in a range from several individual birds to 1,000 birds before 1995 and then increased rapidly thereafter; in 1999, approximately 280,000 live birds were exported; then the exports decreased continuously until 2005. Trade volume of live birds imported to China had been surpassed the export trade volume of live birds from China since 2004.

### Species’ Composition of Live Birds in International Trade

**Figure 2:**
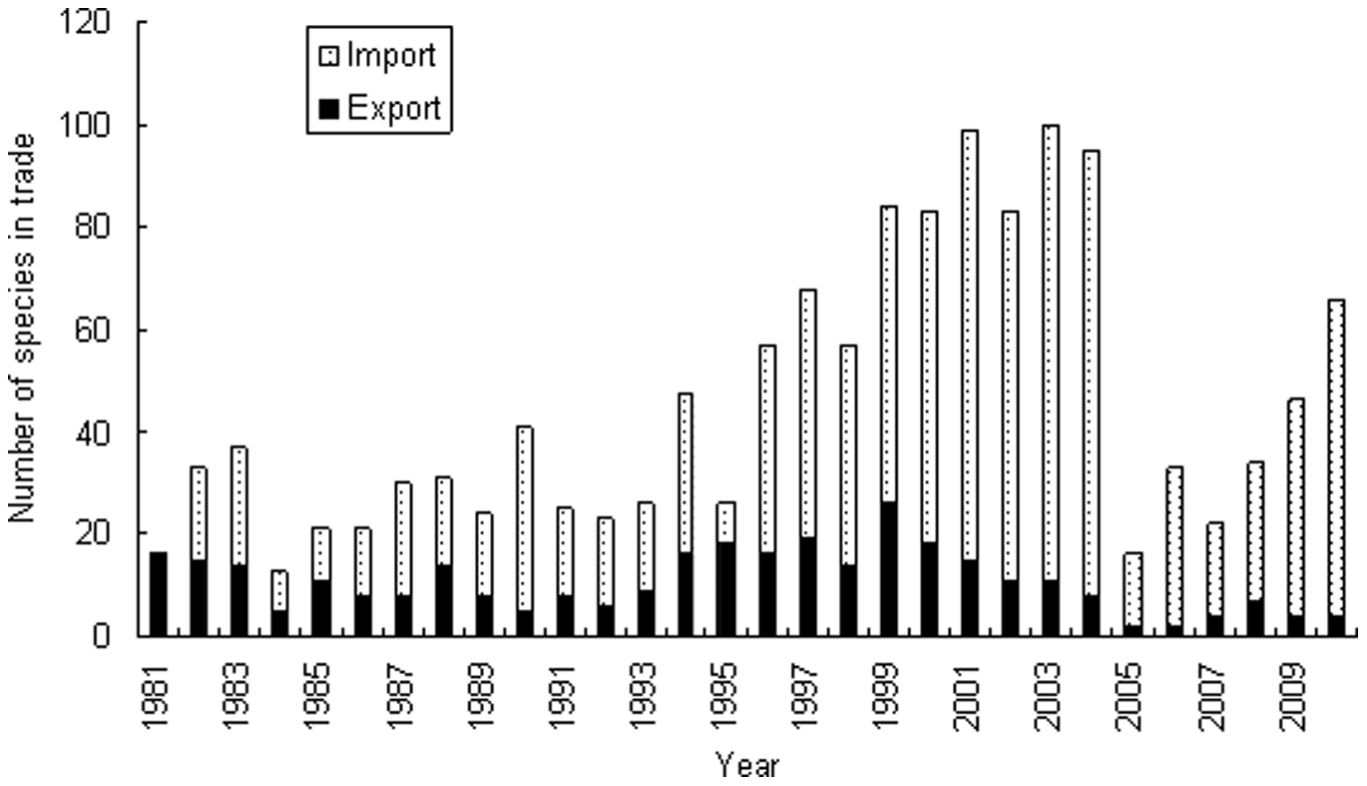
Number of Species of live birds in international trade in China from 1981 to 2010.

Species’ composition of live birds in international trade of China varied from year to year. Different from the import and export quantities of live birds trade of China, more species were imported to China than that exported from China (Figure 2). Number of bird species imported by China increased gradually until 2005, then declined sharply. After then the number of bird species imported by China increased again. Number of bird species exported from China did not change much compared with that of bird import to the country.

During the period from 1981 to 2010, there were nearly 90 bird species exported from China, including birds of Pasittaciformes, Passeriformes, Falconiformes, etc. Parrots and passerines were the most common birds in trade, whereas other birds only accounted for less than 0.3% in the total volume of the bird trade (Table 1). Three significantly traded species of parrots were Fisher’s lovebird (*Agapornis fischeri*), peach-faced lovebird (*Agapornis roseicollis*) and yellow-collared lovebird (*Agapornis personatus*), which accounted for 57.9% of total live birds exported. Major passerine species in exportation were red-billed leiothrix (*Leiothrix lutea*), Java sparrow (*Lonchura oryzivora*), common hill myna (*Gracula religiosa)*, silver-eared mesia (*Leiothrix argentauris*) and black-and-white munia (*Lonchura bicolor*) (Table 2).

**Table 1:** Species’ composition of live birds in international trade of China.

Taxon	Number of Species		Number of live birds (individuals)	
	Export Trade	Import Trade	Export Trade	Import Trade
Anseriformes	2	2	12	351
Ciconiiformes	5	8	100	4,879
Columbiformes	–†	3	–	47
Coraciiformes	–	1	–	2
Cuculiformes	–	5	–	259
Falconiformes	15	13	1397	75
Galliformes	8	18	131	146
Gruiformes	10	9	475	290
Passeriformes	9	3	428,240	33
Piciformes	–	6	–	610
Psittaciformes	31	157	606,185	78,880
Rheiformes	–	1	–	90
Sphenisciformes	1	2	15	317
Strigiformes	6	4	189	14
Struthioniformes	–	1	–	99

† There was no record for live bird trade.

**Table 2:** Major species of live birds exported from China during 1981 to 2010.

Bird Species	CITES Appendices	Number of live birds (individuals)
*Agapornis fischeri*	II	286,050
*Agapornis personatus*	II	117,793
*Agapornis roseicollis* †	II	196,924
*Gracula religiosa*	II	49,047
*Leiothrix argentauris*	II	28,945
*Leiothrix lutea*	II	190,950
*Lonchura bicolor* ‡	III	16,680
*Lonchura oryzivora*	II	128,304

† *Agapornis roseicollis* was deleted from CITES Appendices in 2005. There was no record of the bird exported from China since 2005.

‡ *Lonchura bicolor* was deleted from CITES Appendices since 2007. This bird was exported from China during the period of 2000 to 2002.

There were more than two hundreds species imported to China including birds of Psittaciformes, Ciconiiformes, Piciformes, Anseriformes, etc. Parrots were the mostly imported birds in trade volumes, the same as that in export trade (Table 3). Birds of Ciconiiformes and other species accounted for 5.65% and 2.70% of the total bird imports, respectively (Table 1).

**Table 3:** Major species of live birds imported to China from 1981 to 2010.

Bird Species	CITES Appendices	Number of live birds (individuals)
*Agapornis fischeri*	II	3,571
*Agapornis personatus*	II	7,789
*Agapornis roseicollis* †	II	8,368
*Amazona aestiva*	II	1,965
*Ara ararauna*	II	1,909
*Ara chloropterus*	II	1,767
*Aratinga solstitialis*	II	4,561
*Eos rubra*	II	1,429
*Neopsephotus bourkii*	II	1,056
*Platycercus eximius*	II	1,668
*Poicephalus senegalus*	II	1,659
*Psephotus haematonotus*	II	1,044
*Psittacula krameri* ‡	III	4,648
*Psittacus erithacus*	II	10,335
*Trichoglossus haematodus*	II	2,537
*Phoenicopterus ruber*	II	3,654

† *Agapornis roseicollis* was deleted from CITES Appendices in 2005. There was no record of this bird imported to China since 2005.

‡ *Psittacula krameri* was deleted from CITES Appendices since 2007. There was no record of this bird imported to China since 2005.

### Import Countries and Export Countries of Live Birds in International Trade of China

**Figure 3:**
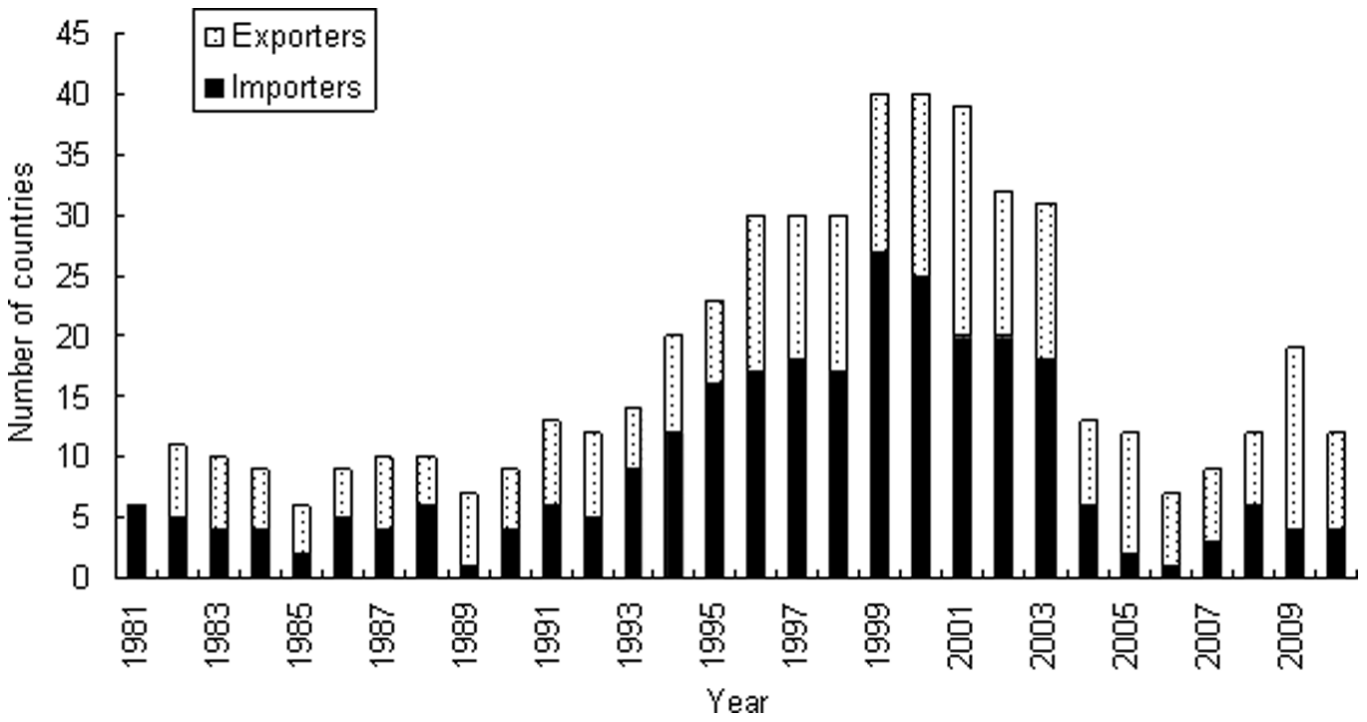
Number of countries in live birds trade with China, 1981–2010.

According to the available records of the CITES Trade Database, import countries and export countries of live birds changed every year (Figure 3). More than 70 countries or regions traded birds with China during the period from 1981 to 2010.

**Figure 4:**
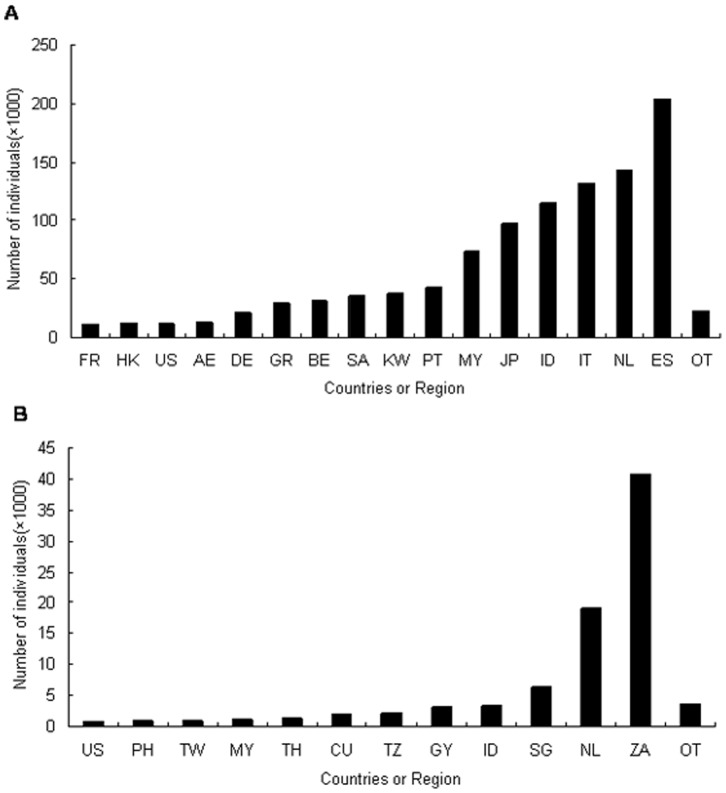
Major importers (a) and exporters (b) of live birds. Major importers (a) and exporters (b) of live birds and the number of individuals trade with China in following countries: AE, United Arab Emirates; BE, Belgium; CU, Cuba; DE, Germany; ES, Spain; FR, France; GR, Greece; GY, Guyana; HK, Hong Kong; ID, Indonesia; IT, Italy; JP, Japan; KW, Kuwait; MY, Malaysia; NL, Netherlands; PH, Philippines; PT, Portugal; SA, Saudiarabia; SG, Singapore; TH, Thailand; TW, Taiwan; TZ, Tanzania; US, United States of America; ZA, South Africa; OT, other countries in trade with China for live birds.

Live birds from China are mainly exported to Europe, South-East Asia, Japan and United States of America (Figure 4a); while most live birds were imported from Africa and South-East Asia to China (Figure 4b). Netherlands, Indonesia, Malaysia and United States of America were major countries traded birds with China, both in export and import. More than 200,000 live individual birds were exported to Spain, which accounted for 19.62% of the whole bird export volume during period of study. About half of the live birds imported by China were from South Africa and most of the birds in trade were parrots.

### Trade Purposes and Sources of Live Birds in International Trade

**Figure 5:**
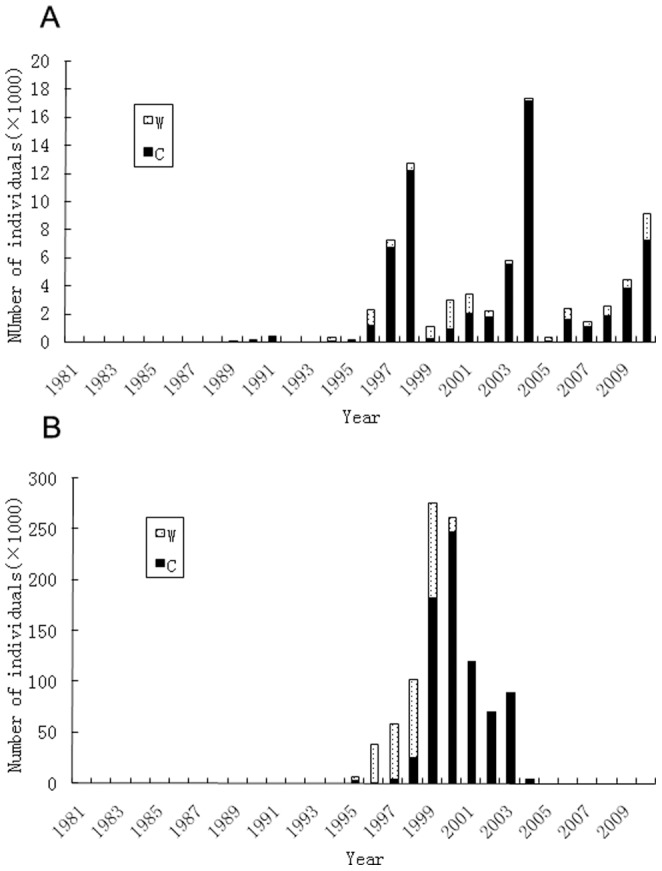
importing (a) and exporting (b) of live birds bred in captivity or caught from wild.

Commercial trade of live birds accounted for 99.8% of the bird export from China while 92% of the birds import by China during the period from 1981 to 2010. Of all bird trades, 6.8% were reported for the purpose of “Zoo” whereas only 1.2% were labeled as for other purposes (Table 4). Live birds were mainly from “Captive-bred” source in both export and import trades while 72% birds imported to China in non-commercial trade were from “Captive-bred” source whereas 82% of birds exported from the country were from “Captive-bred” source (Table 5). Since 2001, most birds exported from China were captive bred (Fig. 5), the percentage of wild caught birds in all birds exported from China decreased from 64% during period of 1991 to 2000 to 24% during the period of 2001 to 2010, the decline was significant (t-test, P = 0.047<0.05). In contrast to the data of bird export, the percentage of wild caught birds in all birds imported by China slightly declined, from 30% during the period of 1991 to 2000 down to 25% during period of 2001 to 2010, the decline was significant (t-test, P = 0.55).

**Table 4:** Number of bird individuals for different trade purposes in China.

Trade Purposes†	Number of individuals	
	Export Trade	Import Trade
B	125	671
E	9	47
H	25	–
N	–‡	27
P	312	115
Q	4	52
S	228	79
T	1,039,864	77,058
Z	970	5,647

Trade purposes are divided into twelve categories: B, Breeding in captivity or artificial propagation; E, Educational; G, Botanical garden; H, Hunting trophy; L, Law enforcement/judicial/forensic; M, Medical including biomedical research; N, reintroduction or introduction into the wild; P, Personal; Q, Circus and traveling exhibitions; S, Scientific; T, Commercial; Z, Zoo.

‡ There was no record for live bird trade.

**Table 5:** Number of bird individuals from different sources in China.

Specimen Sources†	Number of individuals	
	Export Trade	Import Trade
C	754,534	64,820
D	–‡	48
F	27	2323
I	43	92
O	54	40
R	–	1301
U	7	5
W	282,491	11,861

† Specimen sources are divided into nine categories: A, Artificially propagated plants; C, Captive-bred animals; D, Appendix-I animals bred in captivity or Appendix-I plants artificially propagated for commercial purposes; F, Animals born in captivity including F1 and subsequent; I, Confiscated or seized specimens; O, Pre-Convention specimens; R, Specimens of animals reared in a controlled environment; U, Source unknown which must be justified; W, Specimens taken from the wild.

‡ There was no record for live bird trade.

## Discussion

Since the late 1990s, international bird trade in China fluctuated and birds in trade were mostly commercial trade of live birds. After 2003, trade volume, number of bird species and number of countries of bird importation by China exceeded those of bird exported from China. Around the turn of the century, like the trend of international snake trade in China, China once changed from a net export country to a net import country for some snake species since 1990s [14]. When the National Wildlife Management Authority in China imposed a suspension of the trade in snake trades, both imports and exports of all snakes in China have sharply decreased since 2004. The combination of national-level control measures and CITES regulations have controlled the previously unsustainable utilization of snakes in China [15]. Similarly, due to the synergy of CITES and national regulation, the international trade of bird decreased to a low level.

Most live birds were traded as pets in China which were coincided with that of global bird trade [16], [17]. Parrots and passerines in pets trade were common [18], [19], [20], [21], [22], [23], [24]. Lovebirds (*Agapornis* spp.) accounted for the largest proportion of the bird export from China, but their import volume was far less. Almost all lovebirds were captive bred in bird farms in China and traded for commercial purpose as pets. Commercial breed of wild animals was taken as a means of diversifying the animal husbandry in the country. Lovebirds are endemic species to several African countries and used to have a very large range. During late 1990s, Chinese government adopted new policy to cultivate exotic bird species. Then, these parrots were imported to China and were bred commonly in captivity on bird farms in country [25], [26]. The view that breeding of wild animals is an effective way to relieve exploitation pressure on wild populations of those species is still under debate [27], [28], [29], [30]. But there are some successful cases of captive breeding of endangered species for wildlife conservation [31], [32], [33]. Significant export of lovebirds from China does not necessarily threaten wild populations of lovebirds in their natural habitats for only the captive-bred individuals were in international trade. Such commercial breeding of exotic birds for international trade generates revenue to Chinese bird breeders and traders.

Europe, Japan and U.S.A. were major importers of live birds from China. This result is in accord with Nijman’s (2009) research on wildlife trade of South-East Asia [12]. Most parrots imported to China are endemic to tropical African countries, South-East Asia and South American countries. Many wild populations of these bird species were reported declining in their natural habitats thus these birds needed more conservation efforts [34], [35], [36]. African grey parrot (*Psittacus erithacus*) accounted for the largest proportion of birds imported by China. More than three quarters of this bird imported to China were from South Africa. Most of the African grey parrots imported from South Africa by China were captive-bred while the rest imported from other regions were mainly wild-captured, however where the wild populations were still declining due to hunting and other human activities [37], [38]. As one of the main countries for consuming wildlife, China should take more measures to control the import trade of wild-captured birds in the future.

The international trades of black-and-white munia and Java sparrow in China were similar to that of lovebirds, but with no import records of these birds. China is not range country of Black-and-white munia and Java sparrow. Thus, the black-and-white munia and Java sparrow were also captive bred and traded for commercial purpose. Black-and-white munia was described as common in its natural habitats and its population trend was stable; therefore it was deleted from CITES Appendices in 2007 [39]. Main local passerine in trade were red-billed leiothrix, common hill myna and silver-eared mesia, all thees bird species have large geographic ranges. From 1995 to 2000, about 300,000 individuals of red-billed leiothrix, common hill myna and silver-eared mesia were captured from wild and exported from China. There is no report on the population trends of these birds after the significant international trade, though some researchers thought the populations were declining [21], [40], [41], [42]. In order to avoid overexploitation by international trade, these three birds have been listed in CITES Appendix II since 1997. State Forestry Administration of China adopted a policy in 1999: it is forbidden to hunt, to sell or to export birds captured from the wild except for scientific purpose in country [43]. This was why bird export trade volume from China has decreased since 2000.

International wildlife trade is also influenced by wild animal diseases for the wild animal could be reservoirs of unknown pathogen and international trade facilitates the transmission of diseases [44], [45], [46], [47]. During the first few years of 21st century, avian influenza broke out in some Asia countries and had a great impacts on international bird trade [48], [49]. Some of the major trade countries or regions such as European Union and Viet Nam took stricter measures to limit or to stop wild bird trade with other countries [49], [50], [51]. This may be a factor which inluenceed the trade of live birds in China during the period.

More than 40,000 live birds have been imported from South Africa since 1998. 99.7% of the imported birds were for the purpose of commercial trade and 94% of the imported birds were captive bred. In contrast, 83.7% of the birds imported from another country in Africa, Tanzania, were wild-captured and most of the imported birds were for commercial trade. The birds imported by China from Malaysia, Indonesia and Guyana were also mainly from wild source and were traded for commercial purposes. These tropical countries were biodiversity hot-spots with many endemic bird species, the parrots trade of those countries were important in global pets trade [5], [16], [52]. Illegal wildlife trade and poaching were prevalent in these countries [53], [54] while habitats degradation was another threat to local birds in these countries [55]. Now, wildlife mamagement authorities in these countries aware of this situation, and consequently they made a great progress in bird conservation and taking measures to control bird trade, which may be a cause for explaining the decrease of birds imported by China [56], [57], [58], [59].

Trade records of CITES Trade Database have a discrepancy to that of signatory nations of CITES [60], [61]. On the other hand, CITES Trade Database just contains legal wildlife trade information and may be not exactly consistent with the real condition worldwide. The fact that CITES Trade Database relies on country self-reporting and lacks external checks, there may be biased even fake data submitted by some countries for political or economic interests [62], [63]. Due to the prevalence of poaching and illegal trade, the result of our analyses may be conservative compared with actual international trade statistics [59], [64], [65], [66]. However, it still gives us much useful information on CITES listed birds trade in China. Based on the result, we suggest that China should take more measures for better management on wildlife trade: such as to develop methods for identifying the captive-bred and wild animals, carrying out field surveys on wildlife resources and monitoring population dynamics of CITES Appendix listed species, to strengthen CITES law enforcement cooperation with other nations and to encourage more researchers to contribute their expertise in the wildlife trade management.
